# Imaging of Angiotropism/Vascular Co-Option in a Murine Model of Brain Melanoma: Implications for Melanoma Progression along Extravascular Pathways

**DOI:** 10.1038/srep23834

**Published:** 2016-04-06

**Authors:** Laurent A. Bentolila, Roshini Prakash, Daniela Mihic-Probst, Madhuri Wadehra, Hynda K. Kleinman, Thomas S. Carmichael, Bruno Péault, Raymond L. Barnhill, Claire Lugassy

**Affiliations:** 1 California NanoSystems Institute, Los Angeles, CA, 90095 USA; 2 Department of Chemistry and Biochemistry University of California, Los Angeles, CA, 90095 USA; 3 Department of Neurology, David Geffen School of Medicine, University of California, Los Angeles, CA, 90095 USA; 4 Institute of Surgical Pathology, University Hospital Zurich, 8091 Zurich, Switzerland; 5 Department of Pathology and Laboratory Medicine and Jonsson Comprehensive Cancer Center, David Geffen School of Medicine at University of California Los Angeles UCLA, Los Angeles, CA, USA; 6 National Institutes of Health, Bethesda, MD, USA; 7 Orthopedic Hospital Research Center and Broad Stem Cell Center, David Geffen School of Medicine at University of California Los Angeles, Los Angeles, CA, USA; 8 MRC Center for Regenerative Medicine and BHF Center for Cardiovascular Science, Queens Medical Research Institute University of Edinburgh, Edinburgh, UK; 9 Department of Pathology, Institut Curie, and University of Paris Réne Descartes, Paris, France; 10 Department of Translational Research, Institut Curie, Paris, France

## Abstract

Angiotropism/pericytic mimicry and vascular co-option involve tumor cell interactions with the abluminal vascular surface. These two phenomena may be closely related. However, investigations of the two processes have developed in an independent fashion and different explanations offered as to their biological nature. Angiotropism describes the propensity of tumor cells to spread distantly via continuous migration along abluminal vascular surfaces, or extravascular migratory metastasis (EVMM). Vascular co-option has been proposed as an alternative mechanism by which tumors cells may gain access to a blood supply. We have used a murine brain melanoma model to analyze the interactions of GFP human melanoma cells injected into the mouse brain with red fluorescent lectin-labeled microvascular channels. Results have shown a striking spread of melanoma cells along preexisting microvascular channels and features of both vascular co-option and angiotropism/pericytic mimicry. This study has also documented the perivascular expression of Serpin B2 by angiotropic melanoma cells in the murine brain and in human melanoma brain metastases. Our findings suggest that vascular co-option and angiotropism/pericytic mimicry are closely related if not identical processes. Further studies are needed in order to establish whether EVMM is an alternative form of cancer metastasis in addition to intravascular cancer dissemination.

Mortality from cancer is directly related to its invasion and metastatic potential. Metastasis is defined by end points (metastatic lesions detected in specific organs distant from a primary tumor), but the dynamic aspect of the metastatic process to distant organs is still a subject of intense interest in cancer biology^1^. After considerable debate about the potential mechanisms of cancer metastasis continuing until the end of the 19th century, the intravascular dissemination of cancer was finally accepted and is still widely considered as an exclusive paradigm^1^,^2^. Therefore, the interaction of tumor cells with the tumor vasculature is mainly studied for its role in tumor blood supply (tumor angiogenesis) and intravascular metastasis by circulating tumor cells (CTC).

During intravascular dissemination, intravasation at the primary site of the tumor refers to the entry of tumor cells into either the lymphatic or blood circulation. Then, the circulating tumor cells must survive, are passively transported in the circulation, arrest in distant organs, extravasate (escape of cells from the circulation), and grow to form secondary tumors in the new organ environment^1^,^3^.

It has been demonstrated that only a few cells in a primary tumor are able to give rise to a metastasis. This is evident since most cancer cells that leave a solid tumor perish before completing all the steps in the metastatic process. The majority of the CTC never successfully invade a distant organ but die in the vasculature^4^, or perish when the CTC infiltrate distant organs^5^. The complexity of this progression explains, in part, why the metastatic process was suggested to be inefficient^1^.

While the propensity for tumor cells to migrate along anatomic structures, such as nerves and skin appendages has been recognized for many years^6^,^7^ this same capacity of tumor cells to spread along the external vascular surfaces had received almost no attention in the literature until recently. This extravascular tumor spread represents in fact another interaction of tumor cells with vessels in addition to tumor angiogenesis and intravasation. During the past 15 years, two fields of research have emerged which emphasize this interaction: 1. Vascular co-option; 2. Angiotropism and pericytic mimicry. These two processes potentially may be similar; however, both processes have been studied independently and interpreted in entirely different ways. These two different lines of investigation have raised important questions about different aspects of the metastatic process.

Vascular co-option, i.e., the use of pre-existent vessels by tumor cells, was first described in the brain. Indeed, for a long time it has been suggested that glioblastoma can spread via existing vessels rather than being supplied by new ones^8^,^9^. The term “co-opting of vessels” was introduced into the literature by Holash *et al.*^10^ with the observation that “*In contrast with the prevailing view that most tumors and metastases begin as avascular masses, evidence is presented here that a subset of tumors instead initially grows by co-opting existing host vessels.*” Histological examination of vascular co-option by glioma cells have shown organization of tumor cells into cuffs around normal microvessels^11^. More recently, it has been demonstrated that during vascular co-option in lung metastases tumor cells anchor themselves to the endothelial basement membrane of vascular channels^12^.

This research questioned the long-standing concept that all solid tumors are dependent on angiogenesis, i.e., the formation of new vessels, for the sustained growth of tumors and metastasis, which had acquired a central position in cancer research^13^. Therefore, the proposed pathobiological significance of vascular co-option is that tumors may utilize alternative mechanisms in addition to angiogenesis in order to obtain a blood supply. Thus, tumors may initially develop without the need for an angiogenic switch^14^,^15^.

Finally, according to the recent study of Valiente *et al.* another biological role of vascular co-option is to protect cancer cells from death signals generated by plasmin^16^. Using brain colonization assays via intracardiac injection of fluorescent tumor cells (by-passing intravasation of tumor cells), this study demonstrated that tumor cells express plasminogen inhibitors serpins, including serpin B2, in order to promote cancer cell survival and vascular co-option.

In contrast, over the past 15 years Lugassy and Barnhill have drawn attention to angiotropism as a important biological phenomenon, particularly in melanoma^17^,^18^,^19^,^20^. Angiotropism is defined histologically as tumor cells disposed along the external, abluminal surfaces of vessels in a pericytic location without intravasation^17^. Since the first description of angiotropism, it was emphasized that angiotropic melanoma cells were linked to the endothelium by an amorphous basement membrane containing laminin^18^. Angiotropism is optimally recognized either at the invasive front of the tumor or in nearby tissues. Angiotropism promotes pericytic mimicry, the spreading of tumor cells along the abluminal vascular surfaces of microvessels, as demonstrated in different *in vitro* and *in vivo* models^18^,^19^. Using our 3D co-culture model of angiotropism and pericytic mimicry^18^, we also showed that the interaction between melanoma cells and endothelial cells triggered differentially expressed genes associated with cancer progression. Among these genes, 10 were also linked to inflammation, including SERPIN B2. Interestingly, our recent work demonstrated that inflammation promotes angiotropism, pericytic mimicry, and metastasis in a genetically engineered murine melanoma model^18^, confirming again the importance of angiotropism and pericytic mimicry in both melanoma progression and metastasis.

Research on angiotropism has questioned the assumption that all tumor cells use exclusively intravascular dissemination for the formation of metastasis. The main proposed pathobiological significance of angiotropism is that tumor cells have the propensity to spread via continuous migration along the abluminal vascular surfaces or other tracks to nearby or more distant sites, without entering into vascular channels. This alternative mechanism of tumor spread, distinct from intravascular dissemination, has been termed *extravascular migratory metastasis* or EVMM^20^. Current data have shown that tumor cells manifest angiotropism to both microvessels and larger vessels^18^; however the involvement of mature and perfused microvessels versus neovessels still requires further investigation.

In the present work, we have used a murine brain melanoma model, in which tumor cells are injected directly into the brain as a primary site of tumor development. We have analyzed 3D images obtained during the progressive interaction of green fluorescent tagged melanoma cells with red fluorescent lectin tagged vessels. We have investigated the implication of mature vessels versus neovessels in tumor spreading along vessels. We have also examined the expression of Serpin B2 in this murine brain melanoma model, as well as in a series of human melanoma brain metastases. Our goal was to investigate the similarities between vascular co-option and angiotropism, and to discuss the significance of these mechanisms during the metastatic process.

## Results

### Angiotropic dissemination of human melanoma cells in the mouse brain

**Figure 1:**
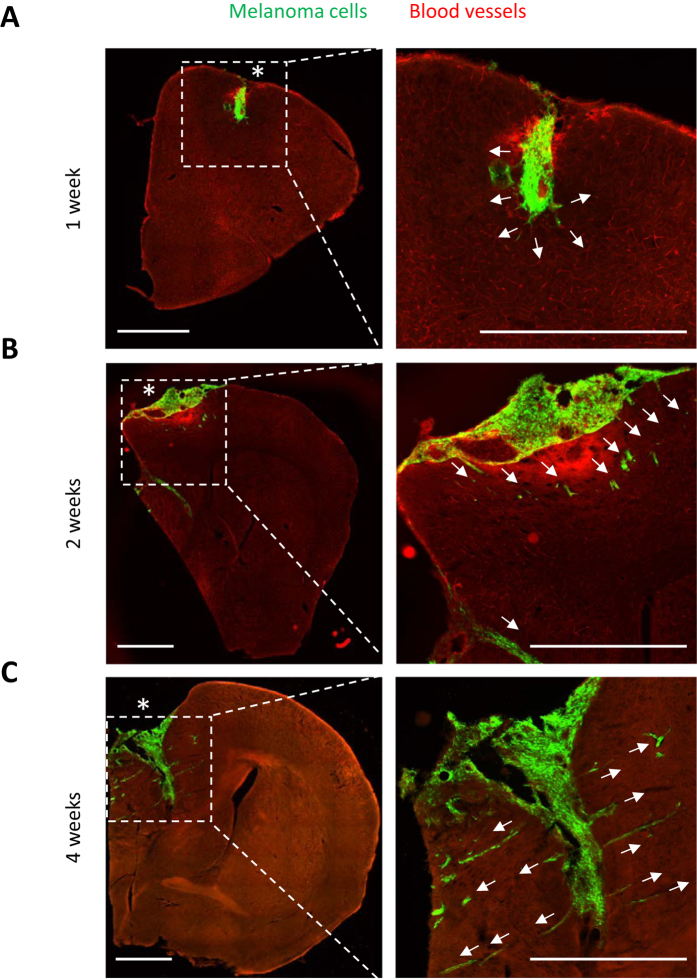
Tumor formation and progressive angiotropic dissemination by human C8161 melanoma cells in the mouse brain. GFP C8161 melanoma cells (green) were stereotaxically injected into the cerebral cortex of Nu/J immuno-compromised mice. The animals were subsequently perfused with Lectin-Dylight 594 (brain microvessels, red) just before being sacrificed at one week (**A**), two weeks (**B**) or four weeks (**C**). The panels on the left show representative coronal sections of one mouse brain hemisphere at these various time points. The panels on the right show fields with increased magnification of the hatched inset areas from the corresponding left panels, with arrows showing angiotropic spread by human melanoma cells. **(A)** After one week, tumor growth of GFP melanoma cells is clearly visible at the injection site (asterisks). Melanoma cells are starting to spread along the abluminal surfaces of the microvascular channels emanating from the tumor mass (right panel, arrows). **(B)** After two weeks, the progressive spread of melanoma cells along the microvascular channels extends far beyond the tumor mass. **(C)** At four weeks, radiating spread of melanoma cells some distances from the injection site along the microvascular channels (arrows) demonstrating strong association with Lectin positive blood vessels (red). White arrows indicate direction of tumor progression. Note that at each time point, melanoma cells were disposed as single cells at the advancing front of the tumor (See also Supplementary Figure S1: tumor growth). Scale bars, 1 mm.

**Figure 2:**
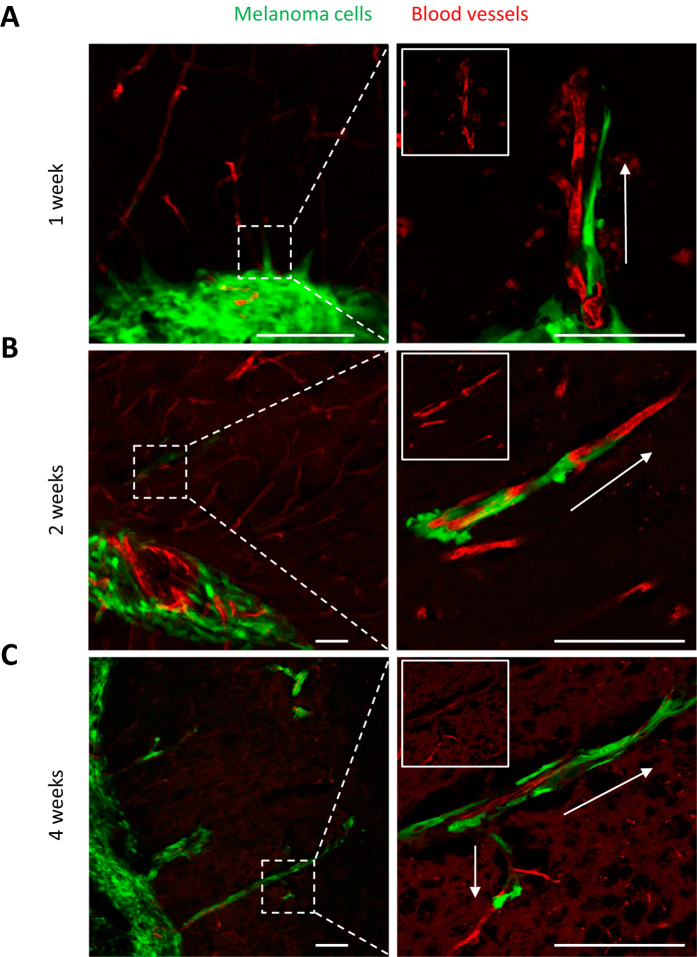
Angiotropism of human melanoma cells in the murine brain tumor. The left panels show representative images of the progression of C8161-induced tumor (green) in coronal sections at one week (**A**), two weeks (**B**) and four weeks (**C**) after injection. The right panels show a magnification of the hatched inset areas from the corresponding left panels. Insets on the right are images of vessels (red) in the absence of the green channels (i.e. the tumor melanoma cells). **(A)** Note initial sprouting of melanoma cells (green) beyond the solid tumor mass as single cells and spreading along the abluminal surfaces of the microvessels (red). This might be termed “the advancing front” of the tumor. On can discern empty vascular lumina with no evidence of intravascular localization of tumor cells. **(B)** Comparable image of GFP-labeled melanoma cells associated with microvascular channels at two weeks. The GFP-labeled melanoma cells are spreading along the abluminal surfaces of the microvessels some distance away from the tumor mass. **(C)** At four weeks, the spreading of melanoma cells has continued some distance beyond from the tumor mass while remaining confined to the external surfaces of endothelial cells. White arrows indicate direction of tumor progression. Scale bars, 100 mm.

**Figure 3:**
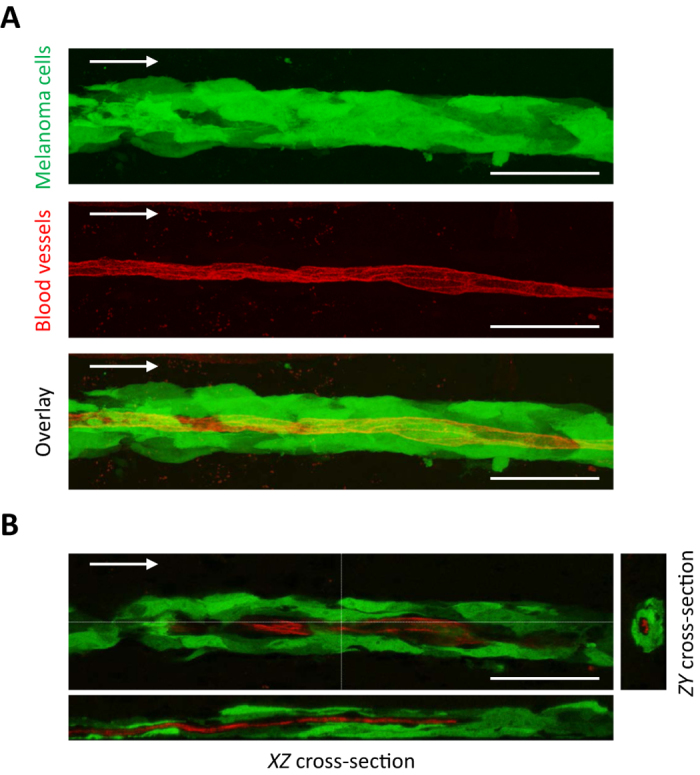
Interactions of tumor cells with the brain microvasculature. **(A)** 2D maximum intensity projections of 70 confocal *z*-stack images for each two separate channels: melanoma cells (top panel, green) and blood vessels (middle panel, red) at four weeks post-implantation. Lower panel: overlay of melanoma cells and blood vessels. Note large groups of elongated melanoma cells enveloping the vascular endothelial surfaces along the vascular plexus. **(B)** A single *z*-slice is shown in the middle panel. The crosshair indicates the position of the *XZ* cross-section and the *ZY* cross-section shown on the two side panels. The melanoma cells are entirely external to the red Lectin-labeled endothelial cells confirming that melanoma cells are arrayed about the abluminal endothelial surfaces and that the intraluminal space does not contain tumor cells (See also Supplementary Movie S1: 3D animation). White arrow indicates direction of tumor progression. Scale bars, 50 mm.

In order to characterize the capacity of tumor cells to spread along external vascular surfaces, we examined early stages of brain tumor formation after injecting human melanoma cells directly into the brain of immuno-compromised Nu/J mice. Examination over time revealed conspicuous tumor growth of the GFP-labeled melanoma cells at the injection site (Fig. 1 and S1). In addition, we observed a striking spread of melanoma cells emanating from the tumor mass. High resolution confocal fluorescence imaging revealed that this tumor spread had a preferential association with the abluminal surfaces of the microvascular channels (the vascular endothelium being clearly delineated by the red fluorescent lectin) from the earliest time point at 1 week to 4 weeks after injection (Fig. 2). While single melanoma cells were seen to elongate along blood microvessels at the advancing front of the tumor (Fig. 2), they formed small clusters or larger groups of cells along the vascular plexus (Figs 1 and 3). These cells exhibited lengthy fine cellular processes extending along the surfaces of the vascular channels. Ramifying vascular channels showed melanoma cells spreading along these branching vessels (Figs 1 and 2). All angiotropic tumor cells were spreading along lectin positive vessels. GLUT1 staining did not reveal additional vessels covered by tumor cells (data not shown).

These results demonstrate the angiotropic nature of the metastatic tumor cell growth which occurs preferentially in close association with the existing brain microvasculature.

### Angiotropic dissemination occurs without evidence of intravasation

We further asked whether the association of tumor cells with vessels far away from the tumor mass could be attributed to tumor cells migrating inside the microvasculature. Figure 3 shows a representative 3D analysis of a large group of elongated melanoma cells wrapped around a blood vessel at four weeks post-implantation. One can clearly observe that all GFP-labeled melanoma cells are exclusively external to the vessels, and that there was no evidence of either intravasation or the presence of intra-luminal tumor cells (Fig. 3B and Supplementary Movie S1).

These results demonstrate that this angiotropic dissemination does not depend on cells circulating through the blood supply but rather must be primarily due to the migration of the tumor cells along the external walls of the brain microvessels.

### Expression of Serpin B2 correlates with tumor cell spreading along microvasculature

**Figure 4:**
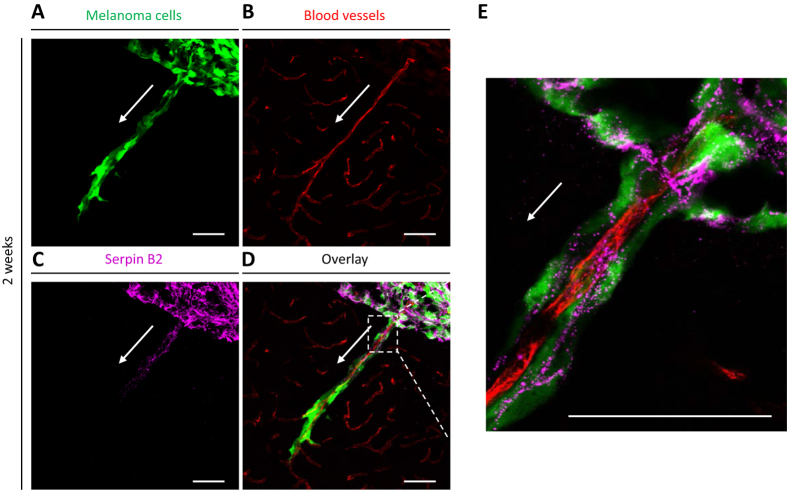
Association of Serpin B2 with human melanoma cells in the murine brain. Immunohistochemistry was performed on coronal sections at two weeks. **(A)** GFP-labeled melanoma cells (green) spreading distally along the abluminal surfaces of the **(B)** microvascular channels (red) from the tumor growth. **(C)** Immunohistochemistry for expression of Serpin B2. **(D)** Overlay of the three images above. **(E)** Magnification of the hatched inset areas from **(D)** showing the strong co-localization of Serpin B2 to the abluminal melanoma cells. White arrow indicates direction of tumor progression. Scale bars, 50 mm.

We then investigated Serpin B2 expression given its important role in cancer survival and brain metastasis^15^. Figure 4 shows striking abluminal expression of Serpin B2 in a pattern largely superimposable with that of GFP-labeled melanoma cells. The merged images showed prominent localization of Serpin B2 to the angiotropic melanoma cells (Fig. 4D,E).

**Figure 5:**
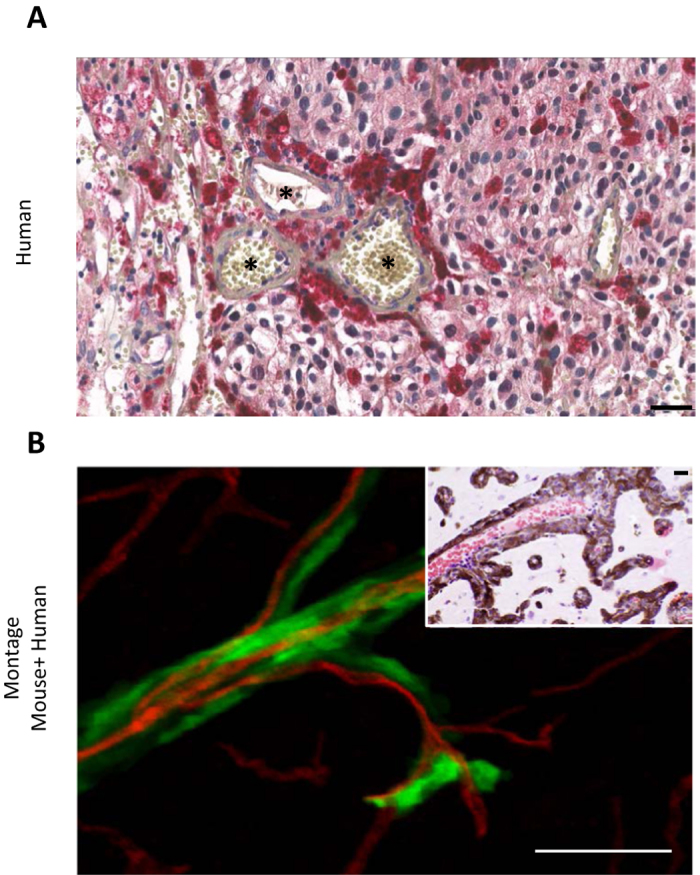
Angiotropism and Serpin B2 in human brain melanoma metastases. **(A)** Human brain melanoma metastasis showing striking expression of SERPIN B2 (dark red) by melanoma cells predominately disposed along the abluminal surfaces (angiotropism) of microvascular channels (Asterisks). **(B)** Montage of mouse brain melanoma spread and metastatic melanoma in the human brain. Lower left image shows GFP-labeled melanoma cells spreading along red lectin-labeled microvascular channels, as shown in the preceding images. Upper right H & E image depicts human melanoma cells disposed along the abluminal surfaces of vascular channels in metastatic melanoma to the brain. The melanoma cells are identified by significant cytoplasmic melanin content and conspicuous cytological atypia. Evidence of intravascular melanoma cells was not identified. Note the conspicuous angiotropism of tumor cells in a pericytic location in both images (green melanoma cells in the murine brain model, melanoma cells containing melanin pigment in the human sample). Scale bars 50 μm.

We further extended our analysis to specimens of human melanoma brain metastases from patients with primary cutaneous melanoma. Among 15 human melanoma brain metastases, 5 showed immunohistochemical expression of SERPIN B2. This expression was observed in an angiotropic distribution focally in all 5 specimens (Fig. 5A). This expression was strong in two of the 5 specimens, and in one of these the pattern of expression was focally diffuse in the melanoma cell population. In contrast, the expression was recorded as relatively weak in 3 cases. It should be noted that some of these 15 brain specimens exhibited some degree of tissue necrosis in areas which precluded complete microscopic interpretation. Moreover, the spatial distribution of tumor spread in the present study is strikingly similar to a previously reported example of human melanoma metastatic to the brain with florid angiotropism^23^ (Fig. 5B).

Altogether, these results validate the clinical relevance of our mouse model and emphasizes the role of Serpin B2 in angiotropic tumor cell migration in the brain.

### Motility of angiotropic melanoma cells in the mouse brain

**Figure 6:**
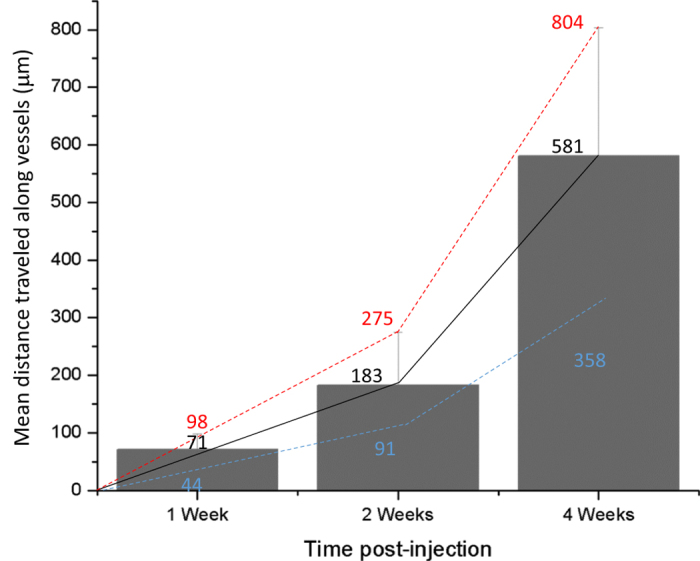
Distances migrated by human melanoma cells in the murine brain. Distances were measured from the margin of the tumor mass to the deepest connected tumor infiltrations spreading along vessels. Data are averages ± SD from n = 2–6 brain slices per each time point, scoring the entire half-hemisphere area, from duplicate experiments. Maximum (red), mean (black) and lowest (blue) distances in mm.

We have further assessed the motility of the angiotropic melanoma cells by scoring the total distance traveled away from the tumor mass over time (Figs 1 and 6). The average migration along vessels observed was 71 μm at one week, 183 μm at two weeks, and 581 μm at four weeks (Fig. 6). No GFP tumor cells were observed neither in the lungs nor in the liver (data not shown).

## Discussion

In the present study, we have observed that melanoma cells implanted in the brain of nude mice spread some distance along the abluminal surface of blood vessels over a period of one to four weeks post-implantation. Importantly, vessels implicated in this tumor progression are functional, preexisting blood vessels, and not newly formed vessels, which is an attribute associated with vascular co-option^14^. Indeed, all tumor cells were associated with lectin-stained vessels, which were in fact perfused vessels with lumina^22^. Furthermore, the images of tumor spread in the present study closely resembled a previously reported example of human melanoma metastatic to the brain with conspicuous angiotropism^23^ (Fig. 5B). The present data also concur with observations from several *in vitro* and *in vivo* models of pericytic mimicry, showing the spread of tumor cells along vessels over time^18^,^19^.

This study also documented perivascular expression of Serpin B2 by angiotropic melanoma cells in the murine brain (Figs 4 and 5). SERPIN B2 was also positive in 33% of the brain melanoma metastases from patients with primary cutaneous melanoma (Fig. 5A). The latter results correspond to the data of Valiente *et al.* in human brain metastases from breast cancer among which 34% of the samples were positive for SERPIN B2^16^. These authors did not describe a specific tissue localization of tumor cells expressing SERPIN B2 in the human samples, while in our study, human brain metastatic melanoma cells positive for SERPIN B2 were clearly angiotropic (Fig. 5). Notably, almost all of these human brain specimens were small partial biopsy samplings without a clear advancing front, where angiotropism is most easily and commonly detected. Also because of the frequent presence of necrosis and tissue ischemia in brain samples, it is possible that SERPIN B2 expression by angiotropic melanoma cells may have been present in a greater number of specimens.

Serpin B2 (also known as plasminogen activator inhibitor type 2 or PAI-2) is a member of the serpin superfamily. The physiological function of Serpin B2 *in vivo* has remained elusive^24^. It has recently been shown that “*by protecting cancer cells from death signals and fostering vascular co-option, anti-PA serpins including serpin B2 provide a unifying mechanism for the initiation of brain metastasis in lung and breast cancers*”^16^. Serpin B2 is also commonly upregulated by inflammation, and a recent study has demonstrated that neutrophilic inflammation promotes angiotropism, pericytic mimicry, and metastatic spread in a genetically engineered melanoma murine model^18^. Interestingly, the interaction between melanoma cells and the abluminal surfaces of endothelial tubules *in vitro* induces significant over expression of Serpin B2, even when plasmin and inflammation are absent^25^.

Although this study involved the brain exclusively as a primary organ for imaging vascular co-option and angiotropism, it is important to emphasize that vascular co-option and angiotropism are observed in many organs affected by different types of primary and metastatic cancers^26^,^27^,^28^,^29^. Finally, several data suggest that resistance and recurrence to current therapies could be linked to angiotropism and vascular co-option^30^,^31^,^32^,^33^.

Taken together, the similarities between vascular co-option and angiotropism/pericytic mimicry strongly suggest that these two phenomena are closely related if not identical.

Indeed, a number of terms and descriptions introduced into the cancer research literature may in fact be closely related processes, rather than representing new or original observations. For example, another closely-related phenomenon described with new terminology and interpretation is that of “*Glioblastoma stem cells generate vascular pericytes to support vessel function and tumor growth*”^34^. In an earlier study entitled “*Pericytic-like angiotropism of glioma and melanoma cells*”, we previously described similar findings emphasizing the migratory nature of these tumor cells in a pericytic manner^30^,^35^,^36^. On the other hand, Cheng *et al.* proposed the role of glioma stem cells as pericyte progenitors, and suggested that they may contribute to the formation of tumor vasculature and the promotion of tumor growth^34^.

Cancer stem cells or cancer cells with stem/embryonic properties are often described as vascular niches, which represents the topography of vascular co-option and angiotropism^37^,^38^. The conspicuous analogies between EVMM and embryonic migration^39^, especially migration of neural crest cells along vessels in the embryo^40^,^41^ suggests that EVMM in melanoma may recapitulate stem/embryonic cell migration in order to reach secondary sites^18^,^33^.

Cancer cells may express a broad spectrum of migratory and invasive properties and are able to modify their migration behaviour in response to changing conditions^42^,^43^,^44^. In the case of migration along vascular tracks, vascular co-option/angiotropism was observed as early as one week in the present brain melanoma model. After four weeks, about 581 μm of brain tissue was traversed by tumor cells migrating along the vascular surfaces. Such distances of travel represent an average velocity of 21 μm per day. Such figures are compatible with a collective migration (0.01–0.05 μm/min)^44^, corresponding to our observations of groups of cells spreading along the external vascular surfaces (Fig. 3).

In addition to angiotropism and vascular co-option, other forms of extravascular migration of melanoma cells, such as invasion of nerves (neurotropism) and skin adnexal structures, i.e., hair follicles and eccrine sweat ducts (adnexotropism), have been recognized for many years^7^. Furthermore, Friedl and co-workers have demonstrated that a variety of anatomic tissue structures may guide the invasion of tumor cells for considerable distances in tissue. Examples include the peritoneum, pleura, and brain ventricles. In addition to vessels and nerves, other structures ensheathed by a basement membrane, such as myofibers and adipocytes, may be used as a scaffolding for cancer cell migration. Other anatomic structures utilized as tracks are collagen fibers, bone cavities, and the choroid plexus and glia limitans in the brain^43^. In the present murine model, no lung or liver metastases were detected. If EVMM occurs in addition to intravascular migration, tumor cells would require more than four weeks to reach such distant sites. This latency in such a vital organ may be one reason why metastases of primary brain malignancies are rarely reported before patient death^45^.

Extravascular migration of tumor cells is generally considered to precede intravasation^42^ or to follow extravasation^16^, and therefore before or after the process of intravascular dissemination of CTC. However, *via* utilization of the different kinds of extravascular routes described above, a continuous migration of melanoma (and other tumors) could constitute an alternative mechanism of tumor spread distinct from intravascular dissemination or EVMM^46^. *Via* this mechanism, tumor cells could spread to nearby, or to more distant sites, without entering vascular channels, representing a veritable alternative metastatic pathway to *“the inefficient intravascular metastatic process”*^1^.

If angiotropism and vascular co-option are similar -if not identical- phenomena demonstrating tumor spread along the abluminal surfaces of vessels, they also represent two very different concepts. In the case of vascular co-option, the uniqueness of the intravascular mechanism of metastasis is implicitly assumed. On the other hand, the concept of angiotropism and pericytic mimicry raises the possibility and likelihood of an alternative mechanism of dissemination. The field of pericytic mimicry and other extravascular forms of tumor cell migration clearly mandates much more research as to the relative importance and/or the reality of extravascular migratory metastasis (EVMM) in melanoma (and other malignancies) progression versus intravascular dissemination. A full understanding of how metastases develop from the primary cancer would have major implications for the design of effective therapies.

## Methods

### Cell line

The C8161 GFP human melanoma cell line^21^ was kindly provided by Dr. Danny Welch (The Kansas University Medical Center).

### Intracranial tumor implantation

#### Ethical Treatment of Animals Statement

This study was carried out in strict accordance with the recommendations in the Guide for the Care and Use of Laboratory Animals of the National Institutes of Health. The protocol was approved by the Animal Research Committee at the University of California, Los Angeles, and all efforts were made to minimize animal suffering.

Six to eight-week-old BALB/c nude female mice were obtained from Charles River Laboratories (Wilmington, MA). In order to implant tumors, mice were anaesthetized using a mixture of isoflurane and oxygen under a steady flow. The mice were then mounted on a stereotaxic frame (Kopf Instruments) with a maintenance dose of anesthesia (2% isoflurane and oxygen). The skin on top of the head was prepared for surgery with alternate applications of alcohol and povidone iodine and then incised along the midline and retracted. Melanoma cells (10^5^ cells/μl in a suspension of L-15 medium) were injected through stereotactic pneumatic injection with Hamilton syringes at a flow rate of 0.25 μl/min. Tumor cells were injected at each of three stereotactic coordinates (anteroposterior (AP) = 1.36, medial lateral (ML)  = 1.07, and dorsal ventral (DV) −0.75) in both hemispheres (six injection per mouse). Before injection the needle was pushed up to 0.5 mm more than the DV coordinate to create a space. At the completion of implantation the needle was drawn out slowly after a delay of 5 minutes. The skin was re-sealed using tissue adhesive, and the animals were allowed to recover. Tumors were allowed to grow for 1, 2, and 4 weeks post injection in three separate cohorts of two mice each, resulting in 12 tumors per time point.

### Visualization of brain vasculature

Before imaging, animals were anaesthetized with ketamine/ xylazine mixture and placed in the supine position and a small incision was made between the neck and shoulder. The jugular vein was exposed for injection of 100 μl of Dylight 594 conjugated Tomato Lectin (1mg/ml) (Vector Laboratories, CA). The lectin was allowed to circulate for 8–10 min after which the animal was sacrificed.

### Tissue processing and immunofluorescence

After whole brain extraction and fixation in 4% paraformaldehyde for 24 h followed by incubation in 30% sucrose in phosphate-buffered saline, 50 μm sections were used for examination with ultra-confocal microscopy. Lungs and livers were also obtained for microscopic examination. For immunofluorescence studies, 50 μm sections were rinsed twice in phosphate buffered saline, pH 7.4 (PBS), and placed in 10 mM sodium citrate buffer, pH 8.5, for 30 min at 80 °C for antigen retrieval. Following 3 brief washes in PBS, sections were then blocked and permeabilized in 10% normal donkey serum containing 0.3% Triton X-100 for 30 min and transferred to MOM block (Vector Labs) for 1 hr at room temperature (RT). After 3 additional washes in PBS, primary antibody cocktails were diluted in blocking solution and left overnight at RT. The following antibodies were used: antibodies against Serpin-B2 at 1:200 (PAI-2 Antibody: sc-6649 Santa Cruz Biotechnology) and Glut-1 at 1:500 (400060-50UG, EMD Millipore) according to the manufacturers’ instructions.

### Fluorescence imaging

Full frame coronal brain slices of 50 μm thickness were imaged with a Leica DM6000 inverted fluorescence microscope (Leica Microsystems, Wetzlar, Germany) equipped with a DFC365 FX CCD camera, green and red filter cubes (Green cube: excitation 450–490 nm BP, dichroic 510 nm, emission 515 nm LP; Red cube: excitation 515–560 nm BP, dichroic 580 nm, emission 590 nm LP) and a 10x/0.3 dry objective (HC PL FLUOTAR 10x/0.3 dry). High resolution imaging was performed by fluorescence confocal microscopy using Leica TCS SP5 and SP8 (Leica Microsystems, Wetzlar, Germany) inverted confocal laser scanning microscopes equipped with a 40x/1.25NA oil objective (HCX PL APO CS 40.0 × 1.25 oil) and a 63x/1.40NA oil objective (HC PL APO CS2 63.0X/1.40 oil). Fluorescent protein and immunofluorescence stains were excited sequentially with visible lasers (GFP: 488 nm, Dylight594: 594 nm, Atto647: 633 nm). Scanning was performed at a line frequency of 400 Hz with an image format of 1024 × 1024 pixels. Each frame was averaged 4 times.

### Image processing

Registration and mosaicking of hundreds of individual images to form one large full frame image of the coronal brain sections was performed with the LAS AF software (Leica Microsystems, Wetzlar, Germany). High resolution images were analyzed, processed and visualized in 3D using the LAS X software (Leica Microsystems, Wetzlar, Germany).

### Measure of the maximum angiotropic migrated distance

Coronal sections at each time point were scored for the presence of tumor cells growing in direct adhesion to the blood vessel exterior walls. Distances were measured from the margin of the tumor mass to the deepest connected tumor infiltration spreading along vessels.

### SERPIN B2 immunostaining of human metastatic melanoma to the brain

This study was carried out in strict accordance with the relevant guidelines and regulations provided by the ethical authorities of the Canton Zurich. All immunohistological experimental protocols were approved by the official ethical authorities of the Canton Zurich (StV 16-2007, Amendment 2014). Informed consent was obtained from all subjects.

Fifteen cases with melanoma metastatic to the brain were retrieved from the archives of the Department of Pathology, University Hospital Zurich. These studies were approved by the Institutional Review Board, UHZ. These specimens were examined for angiotropism and SERPIN B2 expression. Two μm sections were stained with polyclonal rabbit antibody against SERPIN B2 at 1:50 (PAI-2: H-70; Santa Cruz sc-25745). Immunohistochemical staining was performed on automated immunostainer Bond/Leica (Vision BioSystems Ltd, Newcastle Upon Tyne, UK).

## Additional Information

**How to cite this article**: Bentolila, L. A. *et al.* Imaging of Angiotropism/Vascular Co-Option in a Murine Model of Brain Melanoma: Implications for Melanoma Progression along Extravascular Pathways. *Sci. Rep.* **6**, 23834; doi: 10.1038/srep23834 (2016).

## Supplementary Material

Supplementary Information
