# Supplementary material for: Imaging of Angiotropism/Vascular Co-Option in a Murine Model of Brain Melanoma: Implications for Melanoma Progression along Extravascular Pathways
Source: Sci Rep. 2016 Apr 6;6:23834. doi: 10.1038/srep23834 (PMC4822155; doi:10.1038/srep23834)
Supplement: Supplementary Information [file srep23834-s1.pdf]

Title and authors

Imaging of Angiotropism/Vascular Co-Option in a Murine Model of Brain Melanoma. Implications for Melanoma Progression along Extravascular Pathways

Bentolila LA, Prakash R, Mihic-Probst D, Wadehra M, Kleinman HK, Carmichael TS, Péault B, Barnhill RL, Lugassy C

Supplemental Information includes one figure and one movie:

Figure S1

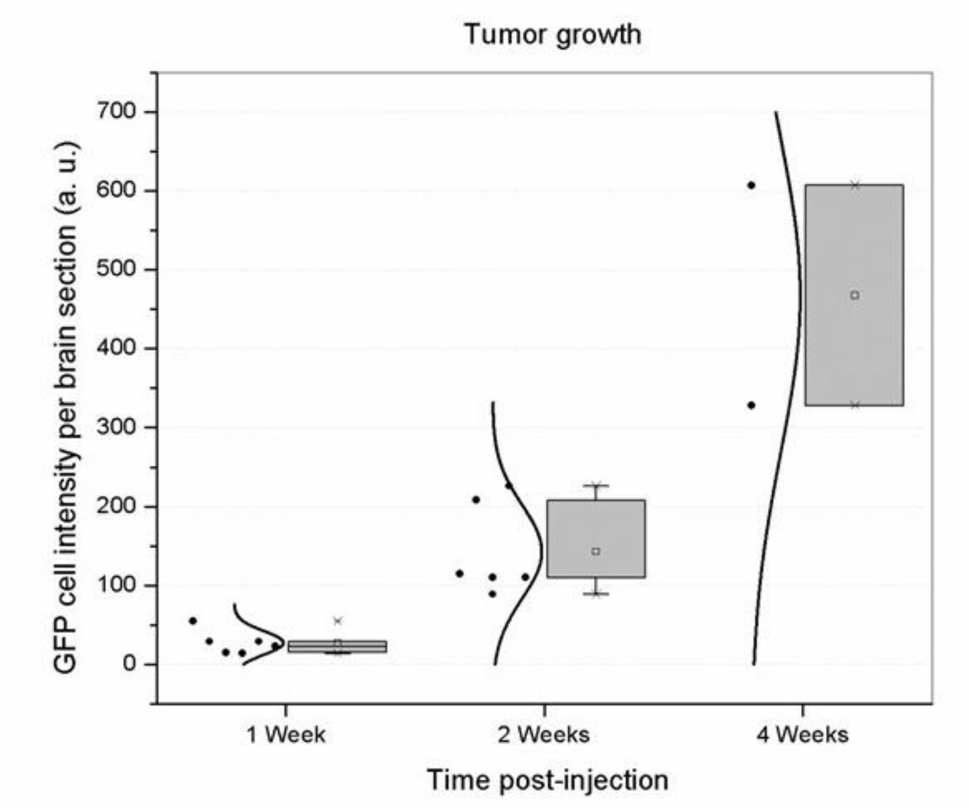

Legend: Quantification of total GFP+ melanoma cells in mouse brain sections from Figure 1. Analysis is from n = 2-6 brain slices per each time point, scoring the entire half-hemisphere area, from at least two independent experiments.

Movie S1.
3D animation of tumor melanoma cell interactions with brain vasculature from Figure 3. The 3D rendering appears with melanoma cells (green) and blood vessel (red) encompassing a volume of 246 μm x 61 μm x 21 μm.

https://ucla.box.com/s/u5ohy7766ka8zqf3beo1skoif1qgnzlq
